# The association of sleep quality, delirium, and sedation status with daily participation in physical therapy in the ICU

**DOI:** 10.1186/s13054-016-1433-z

**Published:** 2016-08-18

**Authors:** Biren B. Kamdar, Michael P. Combs, Elizabeth Colantuoni, Lauren M. King, Timothy Niessen, Karin J. Neufeld, Nancy A. Collop, Dale M. Needham

**Affiliations:** 1 Division of Pulmonary and Critical Care Medicine, David Geffen School of Medicine at the University of California, 10833 Le Conte Ave., Room 37-131 CHS, Los Angeles, CA 90095 USA; 2 Department of Medicine, David Geffen School of Medicine at the University of California, Los Angeles, CA 90095 USA; 3 Outcomes After Critical Illness and Surgery (OACIS) Group, Johns Hopkins University, Baltimore, MD 21205 USA; 4 Department of Biostatistics, Johns Hopkins Bloomberg School of Public Health, Baltimore, MD 21205 USA; 5 Department of Palliative Medicine, Wellspan Health, York Hospital, York, PA 17403 USA; 6 Department of Medicine, Johns Hopkins University, Baltimore, MD 21205 USA; 7 Department of Psychiatry and Behavioral Sciences, Johns Hopkins University, Baltimore, MD 21205 USA; 8 Emory Sleep Disorders Center, Wesley Woods Health Center, Emory University, Atlanta, GA 30322 USA; 9 Division of Pulmonary and Critical Care Medicine, Johns Hopkins University, Baltimore, MD 21205 USA; 10 Department of Physical Medicine and Rehabilitation, Johns Hopkins University, Baltimore, MD 21205 USA

**Keywords:** Sleep, Intensive care unit, Early ambulation, Rehabilitation, Delirium, Sedation

## Abstract

**Background:**

Poor sleep is common in the ICU setting and may represent a modifiable risk factor for patient participation in ICU-based physical therapy (PT) interventions. This study evaluates the association of perceived sleep quality, delirium, sedation, and other clinically important patient and ICU factors with participation in physical therapy (PT) interventions.

**Method:**

This was a secondary analysis of a prospective observational study of sleep in a single academic medical ICU (MICU). Perceived sleep quality was assessed using the Richards-Campbell Sleep Questionnaire (RCSQ) and delirium was assessed using the Confusion Assessment Method for the ICU (CAM-ICU). Other covariates included demographics, pre-hospitalization ambulation status, ICU admission diagnosis, daily mechanical ventilation status, and daily administration of benzodiazepines and opioids via bolus and continuous infusion. Associations with participation in PT interventions were assessed among patients eligible for PT using a multinomial Markov model with robust variance estimates.

**Results:**

Overall, 327 consecutive MICU patients completed ≥1 assessment of perceived sleep quality. After adjusting for all covariates, daily assessment of perceived sleep quality was not associated with transitioning to participate in PT the following day (relative risk ratio [RRR] 1.02, 95 % CI 0.96–1.07, *p* = 0.55). However, the following factors had significant negative associations with participating in subsequent PT interventions: delirium (RRR 0.58, 95 % CI 0.41–0.76, *p* <0.001), opioid boluses (RRR 0.68, 95 % CI 0.47–0.99, *p* = 0.04), and continuous sedation infusions (RRR 0.58, 95 % CI 0.40–0.85, *p* = 0.01). Additionally, in patients with delirium, benzodiazepine boluses further reduced participation in subsequent PT interventions (RRR 0.25, 95 % CI 0.13–0.50, *p* <0.001).

**Conclusions:**

Perceived sleep quality was not associated with participation in PT interventions the following day. However, continuous sedation infusions, opioid boluses, and delirium, particularly when occurring with administration of benzodiazepine boluses, were negatively associated with subsequent PT interventions and represent important modifiable factors for increasing participation in ICU-based PT interventions.

## Background

Prolonged immobility is common in critical illness [1–3] and associated with intensive care unit (ICU)-acquired weakness [4, 5] which, in turn, is associated with functional impairments and poor health-related quality of life after hospital discharge [5–7]. Recent evidence has demonstrated that early mobilization in the ICU is feasible and safe [8–12], and may lead to reductions in delirium [9, 10], duration of mechanical ventilation [9, 13], and ICU length of stay [10, 13–16], along with improved outcomes following hospital discharge [17].

Despite the potential benefits of early mobilization, various patient-related factors have been identified as potential barriers to physical therapy (PT) interventions in the ICU, including higher oxygen requirements, continuous renal replacement therapy, multi-organ dysfunction, and sedating medications [18, 19]. Poor sleep quality, which is common in ICU patients [20], negatively affects physical functioning among those who are not critically ill [21, 22]. This observation has led to speculation that sleep may be an important and modifiable barrier to ICU-based PT interventions [23]; however, this association has not been empirically evaluated. Hence, as part of a prospective ICU quality improvement project [24], we evaluated the association of perceived sleep quality, along with delirium, sedation and other relevant factors, with subsequent participation in PT interventions.

## Methods

### Project setting and design

This analysis examines the association between perceived sleep quality, delirium, sedation and other clinically important patient and ICU factors with patient participation in physical therapy (PT) interventions in the ICU. This secondary data analysis was performed as a part of a multi-faceted sleep quality improvement (QI) project that occurred in the Johns Hopkins Hospital Medical ICU (MICU) over 201 consecutive days from January to July 2010 [24]. At the time of this project, the MICU had 16 private rooms and a 1:2 registered nurse (RN) to patient ratio. Importantly, this MICU has a structured early mobilization program that includes daily monitoring of patients’ status with respect to eligibility for, and receipt of, PT interventions.

The sleep QI project evaluated consecutive patients spending at least one night in the MICU. To evaluate whether sleep quality influenced participation in PT interventions, we evaluated the subset of patients admitted to the MICU with a sleep assessment done immediately prior to a day on which their PT status or MICU disposition was recorded. Only data from patients’ first MICU admission were included.

### Primary outcome: participation in PT interventions

During the study period, all patients were screened for eligibility for PT consultation using a standardized protocol [25]. Within 24 hours of an order for PT consultation, patients began daily assessments by a physical therapist, including an assessment for medical stability that included review of hemodynamic and respiratory status, relevant laboratory data, and interim history [25, 26]. Daily PT interventions were tailored to the level of impairment and activity tolerance of each patient.

PT status was recorded, on a daily basis, as a 5-level categorical variable: (1) eligible for PT and participated in a PT intervention, (2) eligible for PT and did not participate in a PT intervention, (3) ineligible to participate in a PT intervention (e.g., no order for PT, no PT staff available, or patient admitted after 2 pm), (4) discharged from the ICU, or (5) died in the ICU.

### Exposure variables

Perceived sleep quality was assessed daily using the Richards-Campbell Sleep Questionnaire (RCSQ), a validated 5-item questionnaire utilizing a 100-mm visual-analogue scale to evaluate sleep depth, latency, efficiency, quality, and number of awakenings. Higher RCSQ scores represent better sleep and the average of all scores represent overall sleep quality [27]. Each day, all non-comatose, non-delirious patients were asked to complete the RCSQ describing the previous night’s sleep. If the patient was delirious or unable to complete the survey due to communication barriers (i.e., non-English speaking or unable to use a writing instrument), the night shift nurse completed the RCSQ, based on prior studies demonstrating high patient-nurse agreement on the RCSQ in non-delirious patients [28, 29].

Other ICU exposure variables included delirium and coma, which were assessed twice daily by trained MICU nurses using the Confusion Assessment Method for the ICU (CAM-ICU) [30] and Richmond Agitation Sedation Scale (RASS) [31], respectively. Morning delirium status, recorded prior to any attempted PT intervention, was analyzed as a binary variable; if the morning assessment was not recorded, the prior day’s evening assessment was used. Coma was defined as a RASS score of −4 or −5, as in prior research [32].

Additional ICU exposure variables included overnight mechanical ventilation status (binary variable), and administration of infusions and as-needed bolus doses of benzodiazepine and opioid medications on the day prior to attempted PT. Benzodiazepine and opioid infusions were co-administered on 356 of 2020 (18 %) total patient-days, which comprised 95 % of the 373 patient-days (18 % of total patient-days) on which patients received benzodiazepine infusions. Therefore, benzodiazepine and opioid infusions were combined into a single “sedation infusion” variable. Propofol and dexmedetomidine infusions were rarely used in this MICU during the project and, hence, were not evaluated in this analysis. The following baseline demographic and ICU covariates also were included in the analysis: age, gender, race, pre-ICU admission ambulation status, and ICU admission diagnosis category.

### Statistical analysis

Data were summarized using median and interquartile range for continuous variables and proportions for categorical variables, with statistical comparisons of data performed using Wilcoxon rank sum and chi-squared tests, respectively.

We used a first-order Markov model [33] to estimate the probability of participating in PT intervention, not participating in PT intervention, or being ineligible to participate in PT on a given day, and then moving to one of the following five states on the subsequent day: participating in PT intervention, not participating in PT intervention, being ineligible to participate in PT, ICU discharge, or death.

This first-order Markov model was fit using a multinomial regression model, including fixed effects for the PT status on the immediately preceding day and a robust variance estimate to account for within-patient clustering of repeated daily assessments. We were specifically interested in comparing the exposures influencing participation vs. non-participation in PT interventions, among those patients eligible for PT interventions, while treating the other states as competing risks for this analysis. Therefore, in the multinomial model, the reference category for the daily PT status outcome was set to “not participating in PT intervention” and we report the relative risk ratio (RRR) comparing participation in PT vs. not participation in PT, for each of the exposure variables. For the bivariable analysis, each exposure variable was analyzed individually, and for the multivariable analysis, all the exposure variables were included as covariates.

Appropriate modeling of continuous variables was confirmed by evaluating their linear association with the log odds of participation in PT, using a locally weighted scatterplot smoothing (LOWESS) plot [34]. The absence of multicollinearity among variables in the regression model was confirmed using variance inflation factors. Potential statistical interactions between the primary outcome and sleep quality, mechanical ventilation status, administration of benzodiazepines and opioids, and participation in PT interventions on the prior day were assessed by including interaction terms in the Markov model; a statistically significant interaction was noted between delirium and benzodiazepine bolus doses and was included in the final multivariable model.

Additionally, to evaluate for potential rater bias, we performed a sensitivity analysis of the primary regression model including only patient-completed RCSQs. Finally, to compare baseline and intensive care variables stratified by patient- and nurse-completed RCSQs, we used linear, logistic, and multinomial regression for continuous, binary, and categorical variables, respectively; these regression analyses were adjusted for within-patient clustering using a robust variance estimate. A two-sided *p* <0.05 was used to denote statistical significance. All analyses were performed using STATA version 13.1 (StataCorp LP, College Station, TX, USA). An institutional review board (IRB) chair at Johns Hopkins University reviewed this MICU-wide sleep project and determined that it did not require patient consent or full IRB review.

## Results

### Patient demographics and ICU variables

**Fig. 1:**
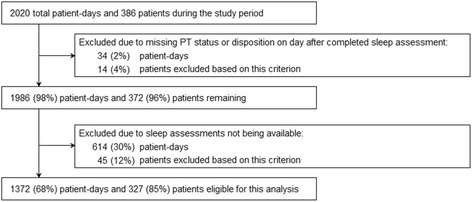
Patient flow diagram. Percentage totals refer to the total number of patient-days and patients during the study period; individual values may not total 100 % due to rounding

During this project, 386 consecutive unique patients were admitted to the MICU, accounting for 2020 patient-days. To examine the association of sleep quality with PT participation, we analyzed a subset of 327 patients, contributing 1372 patient-days, for which there was a valid sleep quality assessment on the night prior to evaluation for PT intervention (Fig. 1).

Comparison of the 327 patients with at least one patient-day included in the multinomial Markov model versus the 59 patients with no eligible patient-days demonstrated no significant differences in age, gender, pre-ICU ambulatory status, admission diagnosis category, average sleep quality, ICU length of stay, and receipt of mechanical ventilation, benzodiazepine boluses, or sedation infusions (Table 1). However, as expected, the patients excluded from this analysis were less likely to ever be delirious, more likely to ever be comatose, and more likely to die in the ICU or hospital.

**Table 1:** Baseline and intensive care variables

Characteristic or exposure	All patients (n = 386)	Excluded from analysis (n = 59)	Included in analysis (n = 327)	*P* value^a^
Baseline variables				
Age, median (IQR), y	55 (44–66)	55 (45–66)	55 (44–66)	0.88
Female sex, n (%)	189 (49)	28 (47)	161 (49)	0.80
Race, n (%)				0.03
White	167 (43)	17 (29)	150 (46)	
Black	197 (51)	36 (61)	161 (49)	
Other	22 (6)	6 (10)	16 (5)	
Ambulatory status prior to ICU admission, n (%)				0.06
Ambulatory	282 (73)	37 (63)	245 (75)	
Not ambulatory	30 (8)	4 (7)	26 (8)	
Unknown/missing	74 (19)	18 (31)	56 (17)	
Intensive care variables				
Admission diagnosis category, n (%)				0.92
Respiratory failure	122 (32)	20 (34)	102 (31)	
Gastrointestinal	58 (15)	7 (12)	51 (16)	
Sepsis, non-pulmonary	47 (12)	7 (12)	40 (12)	
Cardiovascular	39 (10)	5 (8)	34 (10)	
Other	120 (31)	20 (34)	100 (31)	
Average sleep quality, median (IQR)^b^	54 (40–68)	46 (0–73)	55 (40–68)	0.16
Ever received mechanical ventilation, n (%)	220 (57)	39 (66)	181 (55)	0.13
Ever delirious^c^, n (%)	223 (58)	24 (41)	199 (61)	0.004
Ever comatose^c^, n (%)	123 (32)	31 (53)	92 (28)	<0.001
Ever received benzodiazepine bolus, n (%)	93 (24)	10 (17)	83 (25)	0.16
Ever received opioid bolus, n (%)	145 (38)	11 (19)	134 (41)	0.001
Ever received sedation infusion, n (%)^d^	139 (36)	25 (42)	114 (35)	0.27
Length of stay in ICU, median (IQR), d	3 (2–6)	2 (2–6)	3 (2–7)	0.45
Died in the ICU, n (%)	63 (16)	22 (37)	41 (13)	<0.001
Died in the hospital, n (%)	91 (24)	28 (47)	63 (19)	<0.001

*IQR* interquartile range, *ICU* intensive care unit

^a^Calculated using Wilcoxon rank sum for continuous variables, and chi-squared test for categorical variables. Values correspond to “Excluded from analysis” and “Included in analysis”

^b^Richards-Campbell Sleep Questionnaire (RCSQ) consisting of five measures of sleep quality using 100-millimeter visual-analogue scale. Overall sleep quality score calculated by averaging the five sleep quality items on each RCSQ assessment. Higher scores represent better overall sleep quality

^c^As measured using the Confusion Assessment Method for the ICU (CAM-ICU) for delirium, and Richmond Agitation-Sedation Scale (RASS) for sedation. A RASS of -4 or -5 was defined as a comatose state

^d^Includes benzodiazepine and/or opioid infusions. During this study, benzodiazepine infusions were co-administered with opioid infusions on 356 of 373 (95 %) patient-days, and these variables were therefore combined into a single variable

### Patient participation in physical therapy interventions

For days when patients participated in PT interventions, they were eligible to participate on 68 % of subsequent days, with 53 % and 15 % continuing or discontinuing participation, respectively (Table 2). Among days when patients were eligible but did not participate in PT interventions, they participated on 23 % of subsequent days and continued to not participate on 47 % of days.

**Table 2:** Participation in PT and/or PT status on day *t* and *t* + 1^a^

Day *t*	Day *t* + 1^b^						
	Participated in PT, n (%)	Did not participate in PT^c^, n (%)	Ineligible to participate in PT^d^, n (%)	Discharged from ICU, n (%)	Died in ICU, n (%)	Missing, n (%)	Total, n (%)
Participated in PT	**297 (53)**	**84 (15)**	**72 (13)**	**94 (17)**	**2 (0)**	8 (1)	557 (100)
Did not participate in PT^c^	**146 (23)**	**295 (47)**	**57 (9)**	**95 (15)**	**16 (3)**	21 (3)	630 (100)
Ineligible to participate in PT^d^	**91 (42)**	**28 (13)**	**26 (12)**	**68 (31)**	**1 (0)**	5 (2)	219 (100)
No RCSQ assessment^e^	95 (15)	406 (66)	33 (5)	36 (6)	44 (7)	0 (0)	614 (100)
Total	629 (31)	813 (40)	188 (9)	293 (15)	63 (3)	34 (2)	2020 (100)

*PT* physical therapy, *ICU* intensive care unit, *RCSQ* Richards-Campbell Sleep Questionnaire

^a^Bold values highlight the 1372 patient-days included in the multinomial transition model

^b^Row percentage totals may not equal 100 % due to rounding

^c^Including patient-days when patients were unavailable; decreased mental status (due to sedation medications or a primary CNS process); declined PT intervention; were inappropriate (e.g., medically unstable, comfort care orders); or did not require PT interventions (e.g., at functional baseline)

^d^Includes patient-days when order for PT was not placed, PT staff were unavailable, or when patient was admitted to the ICU after 2 pm (too late for PT intervention)

^e^Of 614 patient-days when no RCSQ assessment was performed, patients were ineligible for assessment on 417 (68 %) patient-days due to comatose status. On 197 (32 %) patient-days, the RCSQ was incomplete or not performed

### Sleep quality

When comparing nightly RCSQ ratings prior to patient-days with vs. without participation in PT interventions, there was no significant difference in overall sleep quality (median [IQR] 56 [31–76] vs. 58 [34–75], *p* = 0.69), or in any of the five individual RCSQ sleep quality items.

Moreover, when comparing the 724 patient-completed versus 628 nurse-completed RCSQs, we observed no difference in sleep quality ratings (median [IQR] 58 [33, 78] vs. 57 [35, 75], *p* = 0.82) (Appendix Table 4). Between these two groups, baseline characteristics were generally similar; however, as expected, among intensive care variables, nurse-completed RCSQ assessments were more common while patients were mechanically ventilated, delirious and receiving benzodiazepine and/or opioid infusion (Appendix Table 4).

### Factors related to participation in PT interventions

Our regression analysis demonstrated no association between sleep quality and daily participation in PT interventions (RRR 1.02, 95 % confidence interval (CI) 0.96–1.07 per 10 points on RCSQ, *p* = 0.55) (Table 3); we observed a similar result in a sensitivity analysis including only RCSQs scores completed by patients (RRR 1.05, 95 % CI 0.98–1.14 per 10 points on RCSQ, *p* = 0.15). However, our analysis demonstrated a significant negative association with participation in PT interventions among patients who had received opioid boluses (RRR 0.68, 95 % CI 0.47–0.99, *p* = 0.04), and patients who had received a sedation infusion (RRR 0.58, 95 % CI 0.40–0.85, *p* = 0.01). Additionally, race other than white or black (RRR 0.54, 95 % CI 0.30–0.90, *p* = 0.047) and non-ambulatory status prior to hospitalization (RRR 0.48, 95 % CI 0.27–0.84, *p* = 0.01) were associated with not participating in PT interventions. Finally, we found no association of mechanical ventilation and participation in PT interventions (RRR 1.10, 95 % CI 0.75–1.61, *p* = 0.64).

**Table 3:** Factors associated with daily participation in PT interventions in the ICU

Variable	Bivariable RRR (95 % CI)	*P* value^a^	Multinomial RRR (95 % CI)	*P* value^a^
Overall sleep quality, per 10 points on RCSQ^c^	0.99 (0.94–1.05)	0.77	1.02 (0.96–1.07)	0.55
Baseline variables				
Age, per year	1.00 (0.99–1.01)	0.59	1.00 (0.99–1.02)	0.50
Female sex	0.84 (0.57–1.23)	0.37	0.78 (0.54–1.13)	0.19
Race				
White	REF			
Black	0.76 (0.54–1.06)	0.10	0.84 (0.58–1.23)	0.37
Other	0.72 (0.40–1.32)	0.29	0.54 (0.30–0.99)	0.047
Ambulatory status prior to ICU admission				
Ambulatory	REF			
Not ambulatory	0.83 (0.49–1.40)	0.48	0.48 (0.27–0.84)	0.01
Unknown/missing	0.78 (0.47–1.30)	0.34	0.98 (0.58–1.67)	0.95
Intensive care variables^b^				
Admission diagnosis category				
Respiratory failure	REF			
Gastrointestinal	0.95 (0.50–1.80)	0.86	0.94 (0.49–1.81)	0.86
Sepsis, non-pulmonary	0.87 (0.56–1.36)	0.56	0.78 (0.45–1.36)	0.38
Cardiovascular	1.19 (0.63–2.23)	0.59	1.06 (0.56–2.02)	0.86
Other	1.09 (0.70–1.69)	0.70	1.15 (0.75–1.76)	0.52
Mechanically ventilated	0.93 (0.66–1.32)	0.69	1.10 (0.75–1.61)	0.64
Delirious state^d^	0.50 (0.37–0.68)	<0.001		
Received benzodiazepine bolus dose	1.13 (0.75–1.70)	0.56		
Received opioid bolus dose	0.86 (0.60–1.22)	0.40	0.68 (0.47–0.99)	0.04
Received sedation infusion^e^	0.59 (0.42– 0.81)	0.001	0.58 (0.40–0.85)	0.01
Did not receive benzodiazepine bolus and normal mental status^d,f^			REF	
Received benzodiazepine bolus and normal mental status^d,f^			1.50 (0.88–2.54)	0.13
Delirious state and did not receive benzodiazepine bolus^d,f^			0.56 (0.41–0.76)	<0.001
Delirious state and received benzodiazepine bolus^d, f^			0.25 (0.13–0.50)	<0.001

*PT* physical therapy, *ICU* intensive care unit, *RRR* relative risk ratio, *CI* confidence interval, *RCSQ* Richards-Campbell Sleep Questionnaire

^a^Calculated using a first-order Markov multinomial regression model, with robust variance estimates to account for within-patient clustering of repeated daily assessment of participation in PT interventions. RRR >1 interpreted as having greater participation with PT intervention on the following day

^b^Daily ICU variables measured the day before assessment for participation with PT intervention

^c^Scored using 100-mm visual-analogue scale, with higher scores representing better sleep quality

^d^Measured by the Confusion Assessment Method for the ICU (CAM-ICU) on the morning prior to attempted PT

^e^Includes benzodiazepine and/or opioid infusions. During this study, benzodiazepine infusions were co-administered with opioid infusions on 356 of 373 (95 %) patient-days, and these variables were therefore combined into a single variable

^f^Assessed using interaction term in the multivariable regression model

We observed a statistically significant interaction (*p* = 0.03) between delirium and receipt of benzodiazepines via bolus dosing, necessitating the inclusion of an interaction term in our multinomial model (Table 3). Compared to patients who did not receive a benzodiazepine bolus and had normal mental status, the relative risk of subsequently participating in PT interventions was lower among both delirious patients who did not receive and who did receive benzodiazepine bolus doses (RRR 0.56, 95 % CI 0.41–0.76, *p* <0.001, and 0.25, 95 % CI 0.13–0.50, *p* <0.001, respectively). However, participating in PT interventions did not differ among non-delirious patients receiving vs. not receiving benzodiazepine bolus doses (RRR 1.50, 95 % CI 0.88–2.54, *p* = 0.13).

## Discussion

As part of a prospective QI project to improve sleep in a medical ICU, we evaluated whether perceived sleep quality, delirium, and sedation were associated with subsequent patient participation in PT interventions in the ICU. In our analysis of 327 patients over 1372 ICU days, we found no association between daily perceived sleep quality ratings and participation in PT interventions on the following day. However, sedation infusions, opioid boluses, and delirium in the ICU, particularly when occurring along with benzodiazepine boluses, were strongly associated with patients not participating in PT interventions on the following day.

This analysis was motivated by prior studies in non-ICU patients demonstrating [35–40] lower levels of physical functioning following poor sleep in both chronically ill [22] and elderly adults [21, 41, 42]. These findings suggest that sleep may affect patient participation in physical therapy [43], and that poor sleep might affect ICU outcomes by impairing participation in PT interventions in the ICU setting [23].

In our analyses, we did not observe an association between daily perceived sleep quality and subsequent participation in PT interventions. This result is consistent with a study demonstrating no association between sleep and physical activity in elderly patients in a general medicine unit [44], suggesting that more severe illness, or unmeasured confounders in the inpatient environment, may blunt the positive effects of sleep quality on physical activity. Additionally, we used the RCSQ to measure perceived sleep quality, given its previous validation in the ICU setting [27]. However, prior studies associating sleep and subsequent physical activity utilized other methods to quantify sleep, such as actigraphy [41, 42] and the Pittsburgh Sleep Quality Index [21, 22]. These differences in methodology for sleep measurement may have also contributed to variability in results.

Notably, we found that patients with delirium had decreased participation in PT interventions. This finding builds on prior studies that have observed that patients with greater organ dysfunction and oxygen requirements [19, 45] experience delays in participation in PT interventions. One of these studies also reported delayed participation in PT among patients with deep sedation/coma [19], but was underpowered to detect a similar association in patients with delirium. Hence, this analysis makes an important new contribution in demonstrating an independent negative association of delirium with participating in PT interventions.

Furthermore, our analysis did not reveal an association between receiving mechanical ventilation and participation in PT interventions, contrasting with previous work [19]. Prior studies report that mechanically ventilated patients rarely participate in PT interventions [1, 3] despite evidence that doing so is safe [8–12] and may be beneficial [9, 10, 15]. Our analysis empirically demonstrates that mechanical ventilation alone may not impede patient participation with PT interventions, but sedation and delirium, commonly co-occurring with mechanical ventilation, may be the underlying important barriers to PT interventions [1].

Our findings are consistent with prior research demonstrating that continuous infusions of sedative medications negatively affect delivery of PT interventions in ICU patients [18, 45]. Additionally, our analyses demonstrated a negative association with participation in PT interventions with opioid boluses and with benzodiazepine boluses given in the presence of delirium. Since these bolus medications are often given in the setting of pain and anxiety, future studies should specifically examine the association of these specific symptoms with participation in PT interventions. Nevertheless, our findings support recent clinical practice guidelines suggesting fewer negative consequences of non-opioid and non-benzodiazepine sedation regimens [46], and that the use of sedation medications and delirium may be modifiable barriers to improving rehabilitation in the ICU setting.

Finally, among the baseline variables included in our model, we found that patients with a race other than white or black were less likely to participate in PT interventions. This was an unexpected finding. However, because only 16 (5 %) of the 327 patients in our analysis were neither white nor black, there is little data supporting this finding. On the other hand, as expected, patients who were not ambulatory prior to ICU admission (i.e., those with greater baseline functional impairment) were less likely to participate in PT interventions, possibly highlighting a patient subset with differing requirements or goals for PT interventions in the ICU.

Our analysis has several potential limitations. First, we analyzed participation in PT interventions as a binary variable (i.e., having occurred or not occurred), potentially missing any potential association between sleep quality with the degree of participation in PT interventions (i.e., better sleep quality may have permitted higher level interventions to be performed).

Second, we used the RCSQ to assess sleep quality, as it had previously been validated against polysomnography (PSG) in the ICU setting [27] and was feasible to collect daily in all ICU patients. However, in our study, when patients were delirious or unable to complete the RCSQ, the nurses completed the RCSQ on the patient’s behalf. While prior studies have found high patient-nurse agreement on the RCSQ [28, 29], a separate sub-analysis conducted as part of our sleep QI project found that our MICU nurses tended to overestimate sleep quality on the RCSQ [47], potentially biasing our results. However, a sensitivity analysis including only patient-reported RCSQ scores demonstrated similar findings to our primary results which helps to minimize this concern. Nonetheless, future studies in this field should explore other measures of sleep.

Third, there was missingness in the RCSQ data. However, these missing data represented <10 % of all patient-days included in the analysis, and given that the association of sleep quality with subsequent participation in PT did not trend toward significance, these missing data likely would not have materially changed our overall findings.

Fourth, in evaluating the effect of opioid and benzodiazepine medications, we did not have access to daily drug dosages, thus limiting our ability to evaluate a dose-dependent interaction. Fifth, generalizability of our findings may be limited, as this project involved a single MICU with a pre-existing structured early rehabilitation program [10, 48].

## Conclusions

In this prospective study in a MICU, we did not observe an association between perceived sleep quality and subsequent participation in PT interventions. However, sedation infusions, opioid boluses, delirium, and benzodiazepine boluses (in the presence of delirium) all significantly decreased subsequent participation in PT interventions, representing important modifiable barriers to optimizing patient participation in rehabilitation in the ICU. These findings provide additional rationale and support for ICU clinical practice guidelines recommending minimization of sedating medications and delirium prevention efforts.

## Abbreviations

CAM-ICU, Confusion Assessment Method for the ICU; CI, confidence interval; ICU, intensive care unit; IQR, interquartile range; IRB, institutional review board; LOWESS, locally weighted scatterplot smoothing; MICU, medical intensive care unit; PT, physical therapy; QI, quality improvement; RASS, Richmond Agitation Sedation Scale; RCSQ, Richards-Campbell Sleep Questionnaire; RRR, relative risk ratio

## Appendix

**Table 4:** Intensive care variables by patient- versus nurse-completed RCSQ

Characteristic or exposure	All patient-days with completed RCSQ (n = 1372)	Patient-completed RCSQ (n = 724)	Nurse-completed RCSQ (n = 648)	*P* value^a^
Overall sleep quality, per 10 points on RCSQ^b^	58 (33–77)	58 (33–78)	57 (35–75)	0.82
Baseline variables				
Age, median (IQR), y	55 (45–66)	57 (45–68)	53 (44–65)	0.02
Female sex, n (%)	568 (41)	297 (41)	271 (42)	0.89
Race, n (%)				0.87
White	694 (51)	388 (54)	306 (47)	
Black	517 (38)	235 (32)	282 (44)	
Other	161 (12)	101 (14)	60 (9)	
Ambulatory status prior to ICU admission, n (%)				0.08
Ambulatory	1000 (73)	546 (75)	454 (70)	
Not ambulatory	194 (14)	124 (17)	70 (11)	
Unknown/missing	178 (13)	54 (7)	124 (19)	
Intensive care variables				
Admission diagnosis category, n (%)				0.90
Respiratory failure	660 (48)	346 (48)	314 (48)	
Gastrointestinal	120 (9)	67 (9)	53 (8)	
Sepsis, non-pulmonary	164 (12)	80 (11)	84 (13)	
Cardiovascular	109 (8)	60 (8)	49 (8)	
Other	319 (23)	171 (24)	148 (23)	
Mechanically ventilated, n (%)	873 (64)	402 (56)	471 (73)	0.001
Delirious state^,^ n (%)^c^	550 (40)	60 (8)	490 (76)	<0.001
Received benzodiazepine bolus dose, n (%)	176 (13)	97 (13)	79 (12)	0.67
Received opioid bolus dose, n (%)	276 (20)	168 (23)	108 (17)	0.03
Received benzodiazepine and/or opioid infusion, n (%)	227 (17)	39 (5)	188 (29)	<0.001

*RCSQ* Richards-Campbell Sleep Questionnaire, *IQR* interquartile range, *ICU* intensive care unit

^a^Calculated using linear, logistic, and multinomial regression for continuous, binary, and categorical variables; all regression analyses were adjusted for within-patient clustering using a robust variance estimate

^b^Richards-Campbell Sleep Questionnaire (RCSQ) consisting of 5 measures of sleep quality using 100-millimeter visual-analogue scale. Overall sleep quality score calculated by averaging the 5 sleep quality items on each RCSQ assessment. Higher scores represent better overall sleep quality

^c^As measured using the Confusion Assessment Method for the ICU (CAM-ICU) for delirium

## References

[1] Hodgson C, Bellomo R, Berney S (2015). Early mobilization and recovery in mechanically ventilated patients in the ICU: a bi-national, multi-centre, prospective cohort study. Crit Care..

[2] Berney SC, Harrold M, Webb SA (2013). Intensive care unit mobility practices in Australia and New Zealand: a point prevalence study. Crit Care Resusc.

[3] Nydahl P, Ruhl AP, Bartoszek G (2014). Early mobilization of mechanically ventilated patients: a 1-day point-prevalence study in Germany. Crit Care Med.

[4] Kress JP, Hall JB (2014). ICU-acquired weakness and recovery from critical illness. N Engl J Med.

[5] Fan E, Dowdy DW, Colantuoni E (2014). Physical complications in acute lung injury survivors: a two-year longitudinal prospective study. Crit Care Med.

[6] Fletcher SN, Kennedy DD, Ghosh IR (2003). Persistent neuromuscular and neurophysiologic abnormalities in long-term survivors of prolonged critical illness. Crit Care Med.

[7] Herridge MS, Cheung AM, Tansey CM (2003). One-year outcomes in survivors of the acute respiratory distress syndrome. N Engl J Med.

[8] Bailey P, Thomsen GE, Spuhler VJ (2007). Early activity is feasible and safe in respiratory failure patients. Crit Care Med.

[9] Schweickert WD, Pohlman MC, Pohlman AS (2009). Early physical and occupational therapy in mechanically ventilated, critically ill patients: a randomised controlled trial. Lancet.

[10] Needham DM, Korupolu R, Zanni JM (2010). Early physical medicine and rehabilitation for patients with acute respiratory failure: a quality improvement project. Arch Phys Med Rehabil.

[11] Sricharoenchai T, Parker AM, Zanni JM (2014). Safety of physical therapy interventions in critically ill patients: a single-center prospective evaluation of 1110 intensive care unit admissions. J Crit Care.

[12] Lee H, Ko YJ, Suh GY (2015). Safety profile and feasibility of early physical therapy and mobility for critically ill patients in the medical intensive care unit: beginning experiences in Korea. J Crit Care.

[13] Kayambu G, Boots R, Paratz J (2013). Physical therapy for the critically ill in the ICU: a systematic review and meta-analysis. Crit Care Med.

[14] Engel HJ, Needham DM, Morris PE (2013). ICU early mobilization: from recommendation to implementation at three medical centers. Crit Care Med.

[15] Morris PE, Goad A, Thompson C (2008). Early intensive care unit mobility therapy in the treatment of acute respiratory failure. Crit Care Med.

[16] Lord RK, Mayhew CR, Korupolu R (2013). ICU early physical rehabilitation programs: financial modeling of cost savings. Crit Care Med.

[17] Morris PE, Griffin L, Berry M (2011). Receiving early mobility during an intensive care unit admission is a predictor of improved outcomes in acute respiratory failure. Am J Med Sci.

[18] Thomsen GE, Snow GL, Rodriguez L (2008). Patients with respiratory failure increase ambulation after transfer to an intensive care unit where early activity is a priority. Crit Care Med.

[19] Mendez-Tellez PA, Dinglas VD, Colantuoni E (2013). Factors associated with timing of initiation of physical therapy in patients with acute lung injury. J Crit Care.

[20] Kamdar BB, Needham DM, Collop NA (2012). Sleep deprivation in critical illness: its role in physical and psychological recovery. J Intensive Care Med.

[21] Chien MY, Chen HC (2015). Poor sleep quality is independently associated with physical disability in older adults. J Clin Sleep Med.

[22] Zarrabian MM, Johnson M, Kriellaars D (2014). Relationship between sleep, pain, and disability in patients with spinal pathology. Arch PhysMed Rehabil.

[23] Hopkins RO, Spuhler VJ (2009). Strategies for promoting early activity in critically ill mechanically ventilated patients. AACN Adv Crit Care.

[24] Kamdar BB, King LM, Collop NA (2013). The effect of a quality improvement intervention on perceived sleep quality and cognition in a medical ICU. Crit Care Med.

[25] Korupolu R, Chandolu S, Needham DM (2009). Series on early mobilisation of critically ill patients. Part one: screening and safety issues. ICU Manage.

[26] Hodgson CL, Stiller K, Needham DM (2014). Expert consensus and recommendations on safety criteria for active mobilization of mechanically ventilated critically ill adults. Crit Care.

[27] Richards KC, O’Sullivan PS, Phillips RL (2000). Measurement of sleep in critically ill patients. J Nursing Meas.

[28] Nicolas A, Aizpitarte E, Iruarrizaga A (2008). Perception of night-time sleep by surgical patients in an intensive care unit. Nursing Crit Care.

[29] Frisk U, Nordström G (2003). Patients’ sleep in an intensive care unit--patients’ and nurses’ perception. Intensive Crit Care Nurs.

[30] Ely EW, Inouye SK, Bernard GR (2001). Delirium in mechanically ventilated patients: validity and reliability of the confusion assessment method for the intensive care unit (CAM-ICU). JAMA.

[31] Ely EW, Truman B, Shintani A (2003). Monitoring sedation status over time in ICU patients: reliability and validity of the Richmond Agitation-Sedation Scale (RASS). JAMA.

[32] Watson PL, Pandharipande P, Gehlbach BK (2013). Atypical sleep in ventilated patients: empirical electroencephalography findings and the path toward revised ICU sleep scoring criteria. Crit Care Med.

[33] Meyn S, Tweedie RL (2009). Markov chains and stochastic stability.

[34] Diggle P, Heagerty P, Liang K-Y (2002). Analysis of longitudinal data.

[35] McClain JJ, Lewin DS, Laposky AD (2014). Associations between physical activity, sedentary time, sleep duration and daytime sleepiness in US adults. Prev Med..

[36] Atkinson G, Davenne D (2007). Relationships between sleep, physical activity and human health. Physiol Behav.

[37] Redeker NS, Ruggiero JS, Hedges C (2004). Sleep is related to physical function and emotional well-being after cardiac surgery. Nurs Res.

[38] Tang NK, Sanborn AN (2014). Better quality sleep promotes daytime physical activity in patients with chronic pain? A multilevel analysis of the within-person relationship. PLoS One.

[39] Alessi CA, Yoon EJ, Schnelle JF (1999). A randomized trial of a combined physical activity and environmental intervention in nursing home residents: do sleep and agitation improve?. J Am Geriatr Soc.

[40] Richards KC, Lambert C, Beck CK (2011). Strength training, walking, and social activity improve sleep in nursing home and assisted living residents: randomized controlled trial. J Am Geriatr Soc.

[41] Martin JL, Fiorentino L, Jouldjian S (2010). Sleep quality in residents of assisted living facilities: effect on quality of life, functional status, and depression. J Am Geriatr Soc.

[42] Reyes S, Algarin C, Bunout D (2013). Sleep/wake patterns and physical performance in older adults. Aging Clin Exp Res.

[43] Coren S (2009). Sleep health and its assessment and management in physical therapy practice: the evidence. Physiother Theory Pract.

[44] Beveridge C, Knutson K, Spampinato L (2015). Daytime physical activity and sleep in hospitalized older adults: association with demographic characteristics and disease severity. J Am Geriatr Soc.

[45] Dinglas VD, Parker AM, Reddy DR (2014). A quality improvement project sustainably decreased time to onset of active physical therapy intervention in patients with acute lung injury. Ann Am Thorac Soc.

[46] Barr J, Fraser GL, Puntillo K (2013). Clinical practice guidelines for the management of pain, agitation, and delirium in adult patients in the intensive care unit. Crit Care Med.

[47] Kamdar BB, Shah PA, King LM (2012). Patient-nurse interrater reliability and agreement of the Richards-Campbell sleep questionnaire. Am J Crit Care.

[48] Needham DM, Korupolu R (2010). Rehabilitation quality improvement in an intensive care unit setting: implementation of a quality improvement model. Top Stroke Rehabil.

